# Association between job role and coronavirus disease 2019 (COVID-19) among healthcare personnel, Iowa, 2021

**DOI:** 10.1017/ash.2022.349

**Published:** 2022-12-01

**Authors:** Takaaki Kobayashi, Alexandra Trannel, John Heinemann, Alexandre R. Marra, William Etienne, Oluchi J. Abosi, Stephanie Holley, Angelique Dains, Kyle E. Jenn, Holly Meacham, Barbara A. Schuessler, Linder Wendt, Patrick Ten Eyck, Beth Hanna, Jorge L. Salinas, Patrick G. Hartley, Bradley Ford, Melanie Wellington, Karen B. Brust, Daniel J. Diekema

**Affiliations:** 1 University of Iowa Hospitals & Clinics, Iowa City, Iowa, United States; 2 Hospital Israelita Albert Einstein, São Paulo, Brazil; 3 Institute for Clinical and Translational Science, University of Iowa Hospitals & Clinics, Iowa City, Iowa, United States; 4 Stanford University, Stanford, California, United States

## Abstract

We describe the association between job roles and coronavirus disease 2019 (COVID-19) among healthcare personnel. A wide range of hazard ratios were observed across job roles. Medical assistants had higher hazard ratios than nurses, while attending physicians, food service workers, laboratory technicians, pharmacists, residents and fellows, and temporary workers had lower hazard ratios.

Healthcare personnel (HCP) on the frontlines of the coronavirus disease 2019 (COVID-19) pandemic response risk acquiring infection.^1^ Whether certain job types put HCP at higher risk for acquiring COVID-19 in a healthcare setting remains uncertain. Some studies have reported higher COVID-19 rates in physicians compared with nurses,^2^ and others have reported a higher incidence in nurses.^3^ We investigated the association between COVID-19 infection and job role in the hospital and the incidence of COVID-19 among HCP stratified by vaccine status including long-term (9 months) vaccine effectiveness after a primary series of 2 doses.

## Methods

The University of Iowa Hospitals & Clinics is an 860-bed academic medical center serving as a referral center for Iowa. COVID-19 vaccines became available in December 2020 and vaccine status of HCP was required to be reported to the University Employee Health Clinic (UEHC). HCP were also required to notify the UEHC if they tested positive for severe acute respiratory coronavirus 2 (SARS-CoV-2). We retrospectively collected COVID-19–associated data between January 1, 2021, and September 30, 2021. These data included age, sex, job role, history of COVID-19 vaccination, type of vaccine if applicable, presence of known exposure to a person with COVID-19, history of COVID-19, and if so, the date of positive SARS-CoV-2 test. Job roles were divided into the following categories: nurse, advanced practice provider (APP) including nurse practitioner and physician assistant, attending physician, clerk, custodian, food service, medical assistant, nursing assistant, pharmacist, resident or fellow, researcher, social worker, respiratory therapist, temporary worker, administrative (which included administrative, patient access, coding, finance, and information technology), laboratory, and others. We excluded HCP who were diagnosed with COVID-19 before January 1, 2021, and those whose records indicated only 1 dose of COVID-19 vaccine. Exposure was categorized into one of the following groups: unknown, work, community, or household exposure. HCP were required to wear a medical-grade mask, gown, gloves, and eye protection or face shield when caring for patients with laboratory-confirmed COVID-19 or patients under investigation for COVID-19 until August 19, 2021, when a respirator (ie, N-95) was required.

To plot the cumulative incidence of COVID-19, we used the number of days from January 1, 2021, to SARS-CoV-2–positive test for unvaccinated HCP and the time from 14 days after the second vaccine to the date of positive SARS-CoV-2 test for vaccinated HCP. To calculate the hazard ratio, we used a Cox regression model adjusted for confounding factors including age, sex, exposure, and type of vaccine. We considered vaccine status as a time-dependent variable. In the Cox model, time to disease was measured from the same date (January 1, 2021) for all HCP to control for other time-varying confounders (eg, level of community transmission and prevalence of the delta variant) when comparing the incidence of COVID-19 between vaccinated and unvaccinated HCP. HCP were considered unvaccinated if there was no history of COVID-19 vaccine documented by UEHC or if they had COVID-19 before 14 days passed from the second dose of the vaccine. Vaccine effectiveness was calculated as 100% × (1 − hazard ratio). *P* ≤ .05 was considered statistically significant.

## Results

**Fig. 1.**
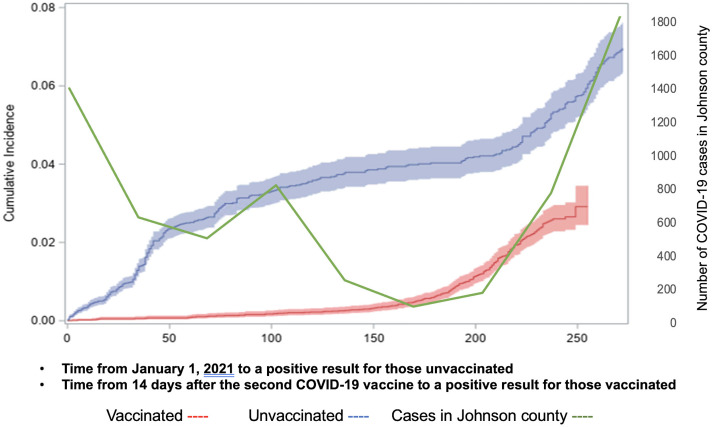
Cumulative incidence with 95% confidential intervals of COVID-19 among healthcare personnel stratified by vaccine status, The University of Iowa Hospitals & Clinics, 2021.

Of 17,101 HCP, 568 (3.3%) were diagnosed with COVID-19 during the study period. The mean age was 39.7 in those with COVID-19 and 40.2 in those without COVID-19 (*P* = .96) (Supplementary Table 1). Among those with COVID-19, 74.9% were female (*P* = .002) and 57% were unvaccinated (*P* = .001). Also, ∼25% were nurses. COVID-19 status varied significantly among different job roles (*P* < .001). Moreover, 848 (5.0%) HCP had known exposure; among them, 124 (14.6%) were diagnosed with COVID-19. Differences in the cumulative incidence between these groups became smaller when community incidence was high, and when 6 months had passed after the second vaccine (Fig. 1).

**Table 1.** Multivariate Cox Hazard Regression Analysis of COVID-19 Among Healthcare Personnel, Iowa, 2021

Parameter	Comparison	Hazard Ratio	95% CI Lower	95% CI Upper	*P* Value^a^
Age	+1 year	0.998	0.992	1.005	.5982
Vaccine type^b^	Unvaccinated		Reference		
	Moderna	0.349	0.227	0.536	**<.0001**
	Pfizer	0.457	0.372	0.560	**<.0001**
Sex	Female vs. male	1.002	0.815	1.231	.9853
Exposure	No known exposure		Reference		
	Work	1.901	0.898	4.022	.0931
	Community	3.632	2.531	5.213	**<.0001**
	Household	7.969	6.264	10.139	**<.0001**
	Nurse		Reference		
Job titles	Administrative	1.093	0.701	1.705	.6945
	APP	0.769	0.359	1.648	.5000
	Attending physician	0.438	0.278	0.690	**.0004**
	Clerk	1.081	0.711	1.645	.7152
	Custodian	0.778	0.427	1.417	.4117
	Food service	0.214	0.053	0.869	**.0310**
	Laboratory	0.155	0.049	0.488	**.0015**
	Medical assistant	2.026	1.379	2.976	**.0003**
	Nursing assistant	0.716	0.435	1.177	.716
	Others	0.713	0.538	0.896	**.0037**
	Administrative	0.735	0.457	1.181	.2034
	Pharmacist	0.376	0.175	0.807	**.0120**
	Residents and fellows	0.352	0.201	0.619	**.0003**
	Respiratory therapist	1.164	0.543	2.467	.6958
	Research	0.838	0.367	1.891	.6616
	Social worker	0.217	0.030	1.551	.1278
	Temporary worker	0.253	0.093	0.688	**.0070**

Note. APP, advanced practice provider; administrative included administrative, patient access, coding, finance and information technology in this study.

a Bold *P* values indicated statistical significance.

b Vaccine status was considered a time-dependent variable. We included HCP who were fully vaccinated (14 d after 2 doses) or unvaccinated. Those with only 1 dose recorded were excluded from the study.

The multivariate Cox hazard regression revealed that being a medical assistant was significantly associated with COVID-19 (hazard ratio [HR], 2.02; 95% CI, 1.38–2.98) (Table 1). HCP with job roles categorized as attending physicians, food service, pharmacy, resident or fellow, temporary, and laboratory were associated with lower COVID-19 incidences. Age and sex were not associated with acquiring COVID-19 (*P* = .27 and *P =* .65). Although work exposure (HR, 1.90; 95% CI, 0.90–4.02) was not significantly associated with COVID-19 compared to unknown exposure, having community exposure (HR, 3.63; 95% CI, 2.53–5.21) and household exposure (HR, 7.97; 95% CI, 6.26–10.14) were significantly associated with acquiring COVID-19. The hazard ratio of the Moderna vaccine was 0.35 (95% CI, 0.23–0.54), suggesting that the 9-month vaccine effectiveness after 2 doses was 65% (95% CI, 45.8%–77.1%). The hazard ratio of the Pfizer vaccine was 0.46 (95% CI, 0.372–0.560), suggesting that the 9-month vaccine effectiveness after 2 doses was 54% (95% CI, 44.0%–62.8%).

## Discussion

Our study revealed that a small proportion of HCP (3.3%) were diagnosed with COVID-19 between January 2021 and September 2021. Among them, ∼25% were nurses. The risk of acquiring COVID-19 varied significantly across job roles. Community exposure and household exposure had significantly higher hazard ratios than work exposure. Receipt of the COVID-19 vaccine was highly protective. However, the difference in cumulative COVID-19 incidence between vaccinated and unvaccinated HCP became smaller over time.

In this study, medical assistants had a higher risk of acquiring COVID-19 than nurses. Although our previous study also demonstrated that being a medical assistant was associated with higher COVID-19 incidence,^4^ individual vaccine information or exposure in the community were not available, and we could not determine whether the higher COVID-19 incidence in medical assistants was associated with lower vaccination rates or exposure outside the hospital. The present study confirmed that the risk of COVID-19 acquisition remains high for medical assistants even after considering vaccine and exposure status. In our healthcare system, medical assistants are responsible for transporting outpatients to rooms, taking an initial brief history, and conducting medication reconciliation; therefore, they have close contact with clinic patients, potentially exposing them to unrecognized COVID-19 cases without the personal protective equipment (PPE) recommended for contact with known or suspected COVID-19 patients.

We initially hypothesized that physician trainees such as residents and fellows and persons working in food service might have a higher risk of acquiring COVID-19. However, they had a lower COVID-19 incidence than nurses. Although aerosol-generating procedures might be a risk factor for SARS-CoV-2 transmission in healthcare settings, respiratory therapists did not have higher risk than nurses, likely due to appropriate PPE.^5^ On the other hand, job roles including pharmacist, temporary workers, and laboratory technicians had an even lower risk of acquiring COVID-19, arguably because they have less patient contact or less varied staff contact. Given that there is high variability in hazard ratios across job roles and nonclinical HCP, such as administrative, had hazard ratios similar to those of nurses, job roles might not be the most important factor for determining the probability of COVID-19 acquisition. In addition, our study confirmed that community exposure and household exposure were associated with a greater risk of SARS-CoV-2 infection than occupational exposure, similar to another recent retrospective study.^6^ However, we did not include the presence of patient care or compliance with PPE, and further prospective studies are needed to determine whether job category plays a significant role for COVID-19 acquisition among HCP.

The short-term vaccine effectiveness was initially high at ∼95%.^7^ However, a study published in early 2022 revealed that vaccine effectiveness decreased to 60%–80% by 7 months after vaccination.^8^ The results of our study are similar in that lower vaccine effectiveness is likely due to waning immune response and new variants such as the SARS-CoV-2 δ (delta) variant. Given that the vaccine protection against COVID-19 decreased over time, we need to ensure that all HCP receive all recommended booster doses after the primary series.^9^

This study had several limitations. We did not include data on whether individual HCP had patient contact. Data regarding whether HCP were using recommended PPE during known exposures were not available. Moreover, though data on socioeconomic status, living conditions, and other factors associated with community and household SARS-CoV-2 exposure risk of HCP may have contributed to our results, that information was not available. Finally, detailed data on levels of masking, vaccination, and test availability in the communities within which our HCP reside were not available for the analysis.

In conclusion, the risk of acquiring COVID-19 varied across job roles, and specific infection prevention strategies might be needed among those with higher risk. Household and community exposure were more significant risk factors than work exposure; HCP should be cautious outside work to prevent exposures. Protection with the primary series of COVID-19 vaccines significantly wanes over time due to declining immunity and with the emergence of new variants. Ensuring that all HCP remain up to date with all vaccine recommendations is critical.

## Supplementary material

For supplementary material accompanying this paper visit https://doi.org/10.1017/ash.2022.349. click here to view supplementary material
