# Supplementary material for: Association between job role and coronavirus disease 2019 (COVID-19) among healthcare personnel, Iowa, 2021
Source: Antimicrob Steward Healthc Epidemiol. 2022 Dec 1;2(1):e188. doi: 10.1017/ash.2022.349 (PMC9726628; doi:10.1017/ash.2022.349)
Supplement: Supplementary file 1 [file S2732494X22003497sup001.docx]

**Supplemental table 1: Characteristics of healthcare personnel stratified by COVID-19 status, Iowa, 2021**

|  | **COVID-19 Positive**  **N = 568** | **COVID-19 Negative**  **N = 16533** | **P value*** |
| --- | --- | --- | --- |
| Age, mean (range) | 39.7 (20-74) | 40.2 (17-97) | 0.96 |
| Gender, female (%) | 412 (74.9) | 10767 (68.6) | **0.002** |
| Vaccine status (%) ** |  |  | **0.001** |
| Moderna | 25 (4.4) | 1605 (9.7) |  |
| Pfizer | 219 (38.56) | 10950 (66.2) |  |
| None | 324 (57.0) | 3978 (24.1) |  |
| Known exposure |  |  | **<0.001** |
| Unknown | 444 (78.2) | 15809 (95.6) |  |
| Work | 9 (1.6) | 159 (1.0) |  |
| Community | 35 (6.2) | 274 (1.7) |  |
| Household | 80 (14.1) | 291 (1.8) |  |
| Job titles (%) |  |  | **<0.001** |
| Administrative | 24 (4.2) | 421 (2.6) |  |
| APP | 7 (1.2) | 212 (1.3) |  |
| Attending physicians | 25 (4.4) | 1371 (8.3) |  |
| Clerk | 27 (4.8) | 531 (3.2) |  |
| Custodian | 13 (2.3) | 345 (2.1) |  |
| Food | 2 (0.4) | 239 (1.5) |  |
| Laboratory | 4 (0.7) | 436 (2.6) |  |
| Medical assistant | 35 (6.2) | 316 (1.9) |  |
| Nurse | 131 (23.1) | 2862 (17.3) |  |
| Nursing Assistant | 18 (3.2) | 542 (3.3) |  |
| Others | 184 (32.4) | 6258 (37.9) |  |
| Patient access/Finance/coding/ IT | 20 (3.5) | 584 (3.5) |  |
| Pharmacist | 7 (1.3) | 447 (2.7) |  |
| Resident/fellow | 15 (2.6) | 956 (5.8) |  |
| Research | 6 (1.6) | 157 (1.0) |  |
| Respiratory therapist | 7 (1.2) | 133 (0.8) |  |
| Social Worker | 2 (0.4) | 107 (0.7) |  |
| Temporary worker | 5 (0.9) | 344 (2.1) |  |

APP: advanced practice provider, IT: Information technology

*Wilcoxon rank-sum test was used for age comparison. Other categorical variables were compared using chi-square.

** We included HCP who were fully vaccinated (14 days after 2 doses) or unvaccinated. Those with only one dose recorded were excluded from the study.
